# High-Performance Au@Ag Nanorods Substrate for SERS Detection of Malachite Green in Aquatic Products

**DOI:** 10.3390/bios13080766

**Published:** 2023-07-28

**Authors:** Xiaoxiao Zhou, Shouhui Chen, Yi Pan, Yuanfeng Wang, Naifeng Xu, Yanwen Xue, Xinlin Wei, Ying Lu

**Affiliations:** 1 College of Food Science and Technology, Shanghai Ocean University, Shanghai 201306, China; 13453949440@163.com; 2 Laboratory of Quality & Safety Risk Assessment for Aquatic Products on Storage and Preservation, Ministry of Agriculture, Shanghai 201306, China; 3 Department of Food Science & Technology, School of Agriculture and Biology, Shanghai Jiao Tong University, Shanghai 200240, China; panyi1018@sjtu.edu.cn (Y.P.); xueyanwen@sjtu.edu.cn (Y.X.); 4 Food Safety Engineering and Technology Research Centre (Shanghai), Shanghai 200240, China; 5 Institute of Food Engineering, College of Life Science, Shanghai Normal University, 100 Guilin Road, Xuhui District, Shanghai 200234, China; yfwang@shnu.edu.cn (Y.W.); xnf1106@shnu.edu.cn (N.X.); 6 Marine Biomedical Science and Technology Innovation Platform of Lingang New Area, Shanghai 201306, China

**Keywords:** Au@Ag nanorods substrate, surface-enhanced Raman scattering (SERS), malachite green

## Abstract

In order to improve the detection performance of surface-enhanced Raman scattering (SERS), a low-cost Au@Ag nanorods (Au@Ag NRs) substrate with a good SERS enhancement effect was developed and applied to the detection of malachite green (MG) in aquaculture water and crayfish. By comparing the SERS signal enhancement effect of five kinds of Au@Ag NRs substrates with different silver layer thickness on 4-mercaptobenzoic acid (4-MBA) solution, it was found that the substrate prepared with 100 µL AgNO_3_ had the smallest aspect ratio (3.27) and the thickest Ag layer (4.1 nm). However, it showed a good signal enhancement effect, and achieved a detection of 4-MBA as low as 1 × 10^−11^ M, which was 8.7 times higher than that of the AuNRs substrate. In addition, the Au@Ag NRs substrate developed in this study was used for SRES detection of MG in crayfish; its detection limit was 1.58 × 10^−9^ M. The developed Au@Ag NRs sensor had the advantages of stable SERS signal, uniform size and low cost, which provided a new tool for SERS signal enhancement and highly sensitive SERS detection method development.

## 1. Introduction

Surface-enhanced Raman Scattering (SERS) has been widely used in the detection and analysis of hazardous substances due to its non-destructive, short-time-consuming, high sensitivity and unique molecular vibration fingerprint [1]. Both the electromagnetic (EM) and chemical (CM) enhancement mechanisms can explain the Raman enhancement effect. It has been found that the electromagnetic enhancement principle of SERS is closely related to surface plasma excitation and the electromagnetic field intensity near the surface, while the chemical enhancement is caused by the charge transfer between molecules and metals [2]. According to the enhancement mechanism, the substrate used for SERS detection had a great impact on the signal strength, and the size, shape and material composition of the substrate would affect the enhancement effect of SERS signal [3,4]. Therefore, a stable, highly bioactive and uniform SERS substrate is crucial for development of sensitive and novel SERS technology.

In the field of SERS detection, metal colloid substrates, flexible substrates and solid substrates have been the most widely used substrates in the last few years [5]. Metal colloid substrates are prepared by reducing the noble metal (Au, Ag, etc.). By controlling the type and concentration of metal salts and reducing agents, the shape and size of various metal colloid substrates can be tailored. The main method for the synthesis of the metal colloid substrates is chemical synthesis, which is simple to operate, easy to synthesize and less time consuming compared with irradiation reduction and laser ablation. Flexible substrates are developed by the deposition of nanoparticles to the flexible base such as paper base, polymer films and adhesive tape [6,7]. Li et al. combined gold nanotriangles and polydimethylsiloxane film to prepare a flexible substrate for rapid CAP detection in food samples [8].

In general, a solid substrate base includes glass slides, silicon wafers, alumina, polyvinyl chloride (PVC) and so on [9,10]. Yu-San Chien et al. utilized alumina beads as a solid base to develop a SERS sensor and demonstrated its SERS sensitivity of 4-aminophenyl disulfide (4-APDS) [11]. However, the use of solid-based and flexible substrates for SERS preparation is associated with tediousness, high cost and it is time-consuming. In contrast, colloidal metal nanoparticles require a simple material synthesis process that is easy to operate and low-cost. Silver nanoparticles (Ag NPs) and gold nanoparticles (Au NPs) are widely utilized due to their surface plasmon resonances in both the visible light and near-infrared regions, rendering them as the most commonly employed metals [12,13]. Muhammad et al. fabricated a SiO_2_@Au composite substrate that enables rapid detection of fipronil residues on egg surfaces, with a lowest detection limit of 0.1 ppm [14]. Chen et al. realized a SERS sensor by combining semiconductors and Ag nanoparticles (NPs) to detect histamine [15].

In addition, the factors such as the size, shape and surface morphology of nanoparticles would have a great influence on the SERS property. Qi et al. [16] utilized gold nanostars to prepare a uniform SERS substrate with PC membrane, which exhibited excellent sensitivity for detecting R6G at concentrations as low as 1 × 10^−10^ M and achieved an enhancement factor of approximately 3.70 × 10^5^. Javad et al. [17] developed gold-aryl nanocubes for SERS analysis, achieving detection limits of 10^−11^ M R6G. Soumya et al. [18] fabricated AgNPr/ZnO NRs substrates and found that the highest SERS signal was obtained from substrates with a 1500 nm nanorod length, highlighting the importance of substrate shape and composition in improving SERS performance.

On the other hand, bimetallic nanoparticles offer the combined benefits of two or more individual nanoparticles. Silver/gold bimetallic nanoparticles not only exhibit the optical enhancement properties of Ag, but also possess the chemical stability of Au, thereby effectively enhancing substrate strengthening ability. Consequently, developing SERS-active substrates based on Ag/Au binary metal nanostructures has become a prominent research topic in SERS. Liu et al. synthesized Au@Ag nanoparticles with varying thicknesses of Ag shell and investigated the correlation between the SERS signal of thiram and the thickness of the silver shell [19]. Chen et al. have developed Au@Ag nanorods (NRs) substrate for detecting TBZ in apple juice and peach juice, achieving LODs of 0.032 ppm and 0.034 ppm, respectively [20]. Au@Ag nanorods possess broader applicability in optical sensing fields due to their ability to combine the advantages of silver’s optical enhancement with their unique shape, compared to Au@Ag nanospheres.

**Scheme 1:**
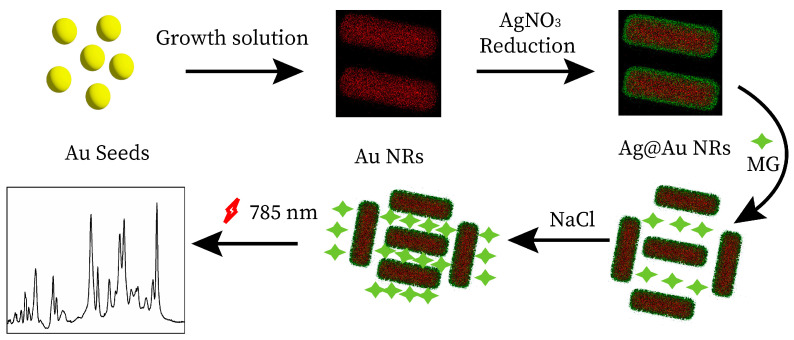
Schematic diagram for the preparation of Au@Ag nanorods substrate and its SERS detection of malachite green.

The objective of this study was to fabricate a highly stable and homogeneous Au@Ag NRs sensor with strong SERS signal. Scheme 1 illustrates the fabrication process of the Au@Ag NRs substrate and its SERS detection mechanism for MG. Initially, Au@Ag NRs substrates were chemically synthesized based on the seed growth method by controlling Ag deposition over Au seeds. Subsequently, we investigated the effect of silver layer thickness on the SERS enhancement of Au@Ag NRs using 4-MBA. The fabricated Au@Ag NRs sensor exhibited significant improvement in SERS signal. Finally, detection of MG in aquatic products was performed to discuss the application feasibility of the fabricated Au@Ag NRs substrate.

## 2. Materials and Methods

### 2.1. Reagent

Cetyltrimethylammonium bromide (CTAB), cetyltrimethylammonium chloride (CTAC), sodium oleate (NaOL), chloroauric acid (HAuCl_4_·4H_2_O), silver nitrate (AgNO_3_) and hydrochloric acid (HCl) were obtained from Sinopharm Chemical Reagent Co., Ltd. (Shanghai, China). L-Ascorbic acid (L-AA), 4-mercaptobenzoic acid (4-MBA) and sodium borohydride were obtained from Qiaoyi Biotechnology Co., Ltd. (Shanghai, China). Malachite green, 2,3-dichloro-5,6-dicyano-1,4-benzoquinone and other chemicals were purchased from Aladdin Industrial Co., Ltd. (Shanghai, China). Ultrapure water was used in all experiments from Shanghai Ding-shuo’s water purification system (Shanghai Ding-shuo Electronic Technology Co., Ltd., Shanghai, China), which was deionized and ultra-purified to 18.2 MΩ.

### 2.2. Synthesis of Au@Ag NRs Substrate

The Au@Ag NRs substrate was fabricated on the basis of the method of Pu H et al. with a slight modification [21]. Firstly, AuNRs were synthesized by the seed-mediated approach. Briefly, 0.01 M NaBH_4_ was rapidly added to the solution by mixing 9.75 mL 0.1 M CTAB solution with 25 μL 0.01 M HAuCl_4_. The solution, after incubation for 2 h at 37 °C, was used as the Au seed. Next, 30 mL solution containing CTAB (0.768 g) and NaOL (0.547 g) was mixed with 1.35 mL 0.01 M AgNO_3_, stirred for 5 min and incubated at 37 °C for 15 min. Then 30 mL 1 mM HAuCl_4_ was added to the above solution and stirred at 700 rpm for 90 min. After the solution turned colorless, 1 M HCl was added and the speed was adjusted to 400 rpm for 15 min. Then, 1 mM ascorbic acid solution was added while stirring vigorously for 30 s. Finally, 100 μL of Au seed solution was added to the solution and kept incubated for 15 h at 37 °C, to grow AuNRs. The synthesized AuNRs were centrifuged twice at 8000 rpm (15 min) and resuspended with 30 mL ultra-water for the next use.

Next, Ag was modified on the surface of AuNRs. Different volumes of 0.01 M AgNO_3_ (50, 100, 200, 300, 400 μL) were mixed with 100 μL 0.08 M CTAC and incubated for 10 min at 37 °C. Then, 2.5 mL of AuNRs were added and incubated at 37 °C for 5 min. Subsequently, 100 μL 0.1 M ascorbic acid was added to the solution while shaking vigorously. After the solution reacted for 4 h at 37 °C, the resultant solution was purified through centrifugation twice at 7000 rpm (15 min) and 4600 rpm (15 min), and resuspended with 3 mL ultra-water for the next use.

### 2.3. Characterization of Au@Ag NRs Substrate

The UV spectra of AuNRs and Au@Ag NRs were acquired using a UV-Vis spectrophotometer (Beijing Puxi General Instrument Co., Ltd., Beijing, China). Transmission electron microscopy (TEM) was utilized to obtain TEM images of the aforementioned nanoparticles with Talos L120C G2 from FEI NanoPorts Co., Hillsboro, OR, America being employed for this purpose. The HAADF-STEM images were acquired by utilizing the Talos F200X (FEI NanoPorts Co., Hillsboro, OR, USA), a high-angle annular dark-field scanning transmission electron microscope.

### 2.4. SERS Detection Based on Au@Ag NRs Substrate

SERS spectra were acquired using a portable Raman spectrometer, the BWS465-785s from BWTEK in Shanghai, China. The SERS enhancement of the synthetic Au@Ag NRs substrate was evaluated using 4-mercaptobenzoic acid (4-MBA) standard. Briefly, 10 μL10^−3^ M 4-MBA was mixed with 1000 μL synthetic AuNRs and Au@Ag NRs. After incubation for 10 min, 10 μL mixture was dropped on the glass slide for SERS detection. Then, different concentrations of 4-MBA (10^−7^ M, 10^−8^ M, 10^−9^ M, 10^−10^ M, 10^−11^ M) were detected to analyze the SERS detection sensitivity. The parameter settings of the instrument were excitation wavelength (785 nm), power (50%), integration time (10,000 ms) and accumulation (3 times).

Detection of MG based on Au@Ag NRs substrate were performed as following. Firstly, 100 μL of different concentrations of MG solution (5 × 10^−9^ M~2 × 10^−7^ M) was mixed with 100 μL Au@Ag NRs substrate and 30 μL of 0.1 M NaCl, respectively. Subsequently, 10 μL of the mixed solution was dropped on the glass slide for detection after incubation for 10 min. The parameters of SERS detection are the same as the 4-MBA standard test, except for the power (100%).

### 2.5. Detection of Real Samples Based on Au@Ag NRs Substrate

The real sample was prepared following the method of Xu T et al. with slight modification [22]. Initially, 2 g of sample homogenate was mixed with 500 μL NH_2_OH-HCl solution. Subsequently, 10 mL acetonitrile, 1g anhydrous magnesium and 4 g alumina were vigorously added and mixed. The supernatant was dried with nitrogen at 50 °C after centrifugation (4000 rpm, 5 min) and the residues were dissolved in 2,3-dichloro-5,6-dicyano-1,4-benzoquinone solution. Subsequently, alumina was added and mixed well and the mixture was centrifuged (12,000 rpm, 10 min). Finally, the supernatant was filtered through a 0.22 μm filter. Then, the real samples were prepared by mixing the MG standard solution with the above solution.

### 2.6. Statistical Analysis

Detection results were presented as mean value and standard deviation (*n* = 3), and Raman spectra were baseline corrected and smoothed by the BWTEK program. The limit of detection (LOD) for MG was determined by the following formula:

LOD = 3S_b_ + Y_b_

S_b_, above, is the standard deviation of the SERS intensity at 1617 cm^−1^ and Y_b_ is the mean intensity of the blanks at 1617 cm^−1^.

## 3. Results and Discussion

### 3.1. Characterization of Au@Ag NRs Substrate

**Figure 1:**
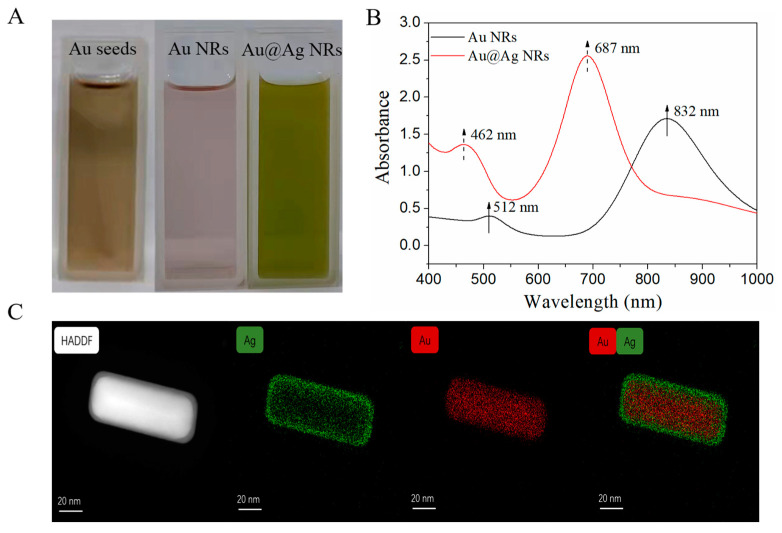
(**A**) Images of Au seed solution, Au NRs solution and Au@Ag NRs solution; (**B**) The UV spectra of AuNRs and Au@Ag NRs substrates; (**C**) A HADDF-STEM image of Au@Ag NRs (left); elemental mapping of Ag, Au and both elements of Au@Ag NRs substrate.

In this study, Au@Ag NRs substrate were fabricated by three steps, which were the synthesis of Au seeds, Au NRs and Au@Ag NRs. After the successful preparation of each step, the optical properties of each product change [23]. The colors of Au seeds, Au NRs and Au@Ag NRs changed from brown, to light red, then to green (Figure 1A), which was consistent with the report of Pu et al. [21]. It has been shown that Au@Ag NRs substrates have been successfully fabricated. In addition, a clear wavelength shift of the AuNRs and Au@Ag NRs substrates was observed in the UV spectrophotometry (Figure 1B). When the AuNRs were covered with Ag, the longitudinal plasmon resonance had a blue shift from 832 nm to 687 nm. When the longitudinal plasmon resonance wavelengths was near 690 nm, the Au@Ag NRs substrate was green [24]. Hence, the blue shifts of the longitudinal plasmon resonance wavelengths was consistent with the color change of AuNRs and Au@Ag NRs. It was found that when Ag was modified to the surface of AuNRs, Ag had stronger plasma absorption characteristics and a shorter plasma resonance wavelength, leading to the blue shift [25]. The wavelengths of the transverse plasmon resonance also showed a blue shift from 512 nm (AuNRs) to 462 nm (Au@Ag NRs) (Figure 1B). These results proved that Au@Ag NRs was successfully synthesized.

In addition, HAADF-STEM and EDS were employed to achieve atomic-scale realization of the construction of Au@Ag NRs substrate. As depicted in Figure 1C, a distinct core-shell structure (grey shell and light core) was observed by HADDF. This is attributed to the difference in atomic number of Au and Ag [26]. Furthermore, in EDS analysis, different colors represent different elements, with red representing Au and green representing Ag. EDS elements also demonstrated the successful synthesis of Au@Ag NRs substrate.

### 3.2. SERS Enhancement Evaluation of Au@Ag NRs Substrates with Different Silver Thickness

**Figure 2:**
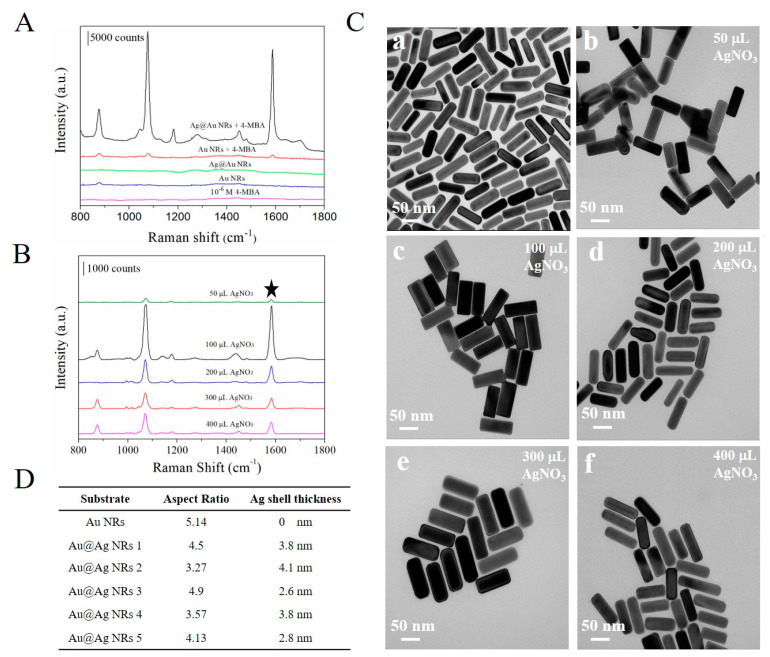
(**A**) SERS intensity of 4−MBA using AuNRs and Au@Ag NRs substrates. (**B**) SERS spectra of 4−MBA based on Au@Ag NRs substrate fabricated with varying amounts of AgNO_3._ The black star refers to utilize the SERS intensity at 1581 cm^−1^ to assess the amounts of AgNO_3_. (**C**) TEM images of Au NRs (**a**) and Au@Ag NRs fabricated with varying amounts of AgNO_3_; (**b**) 50 μL, (**c**) 100 μL, (**d**) 200 μL, (**e**) 300 μL, (**f**) 400 μL. (**D**) The aspect ratio and thickness of the Ag shell of Au@Ag NRs substrates fabricated with varying amounts of AgNO_3_.

The SERS enhancement capability could be affected by the thickness of the Ag shell in Au@Ag NRs substrates [20]. Therefore, Au@Ag NRs substrates were fabricated by adding varying AgNO_3_ amounts and evaluated for their ability to enhance SERS signals. As shown in Figure 2A, 4-MBA standard solution was detected by AuNRs and Au@Ag NRs substrate. With 10^−6^ M 4-MBA standard solution, AuNRs and Au@Ag NRs substrates were detected individually, with which no peaks appeared. This indicated that the presence of AuNRs and Au@Ag NRs substrates does not affect SERS detection. The addition of 4-MBA to Au NRs substrates could enhance the peaks at 1078 cm^−1^ and 1581 cm^−1^, which belong to the circular breathing pattern and C=N stretching, respectively [27]. While the addition of 4-MBA to Au@Ag NRs substrate, the SERS intensity of peaks at 1078 cm^−1^ and 1581 cm^−1^ was significantly enhanced. The SERS intensity of 4-MBA at 1581 cm^−1^ indicated an increase in SERS intensity by a factor of 8.7 when using Au@Ag NRs substrate instead of AuNRs substrate. The Au@Ag NRs substrate exhibits a higher enhancement effect on 4-MBA, which can be attributed to the stronger bonding between Ag and S in 4−MBA [28].

Furthermore, in Figure 2B, the SERS intensity of Au@Ag NRs with varying silver thickness was compared. It was observed that the highest SERS intensity for 4−MBA detection was achieved by Au@Ag NRs substrates fabricated using 100 μL AgNO_3_. The volume of AgNO_3_ used during the preparation process can affect the aspect ratio, Ag shell thickness and morphology of Au@Ag NRs substrates [29,30]. As shown in the TEM results (Figure 2C), homogeneous and equal morphology was obtained when preparing Au@Ag NRs using 100 μL AgNO_3_. As shown in Figure 2D, Au@Ag NRs prepared using 100 μL AgNO_3_ had the smallest aspect ratio (3.27) and the thickest Ag layer (4.1 nm). Therefore, Au@Ag NRs prepared using 100 μL AgNO_3_ were selected for subsequent experiments.

### 3.3. SERS Detection Sensitivity of 4-MBA Based on Au@Ag NRs Substrate

**Figure 3:**
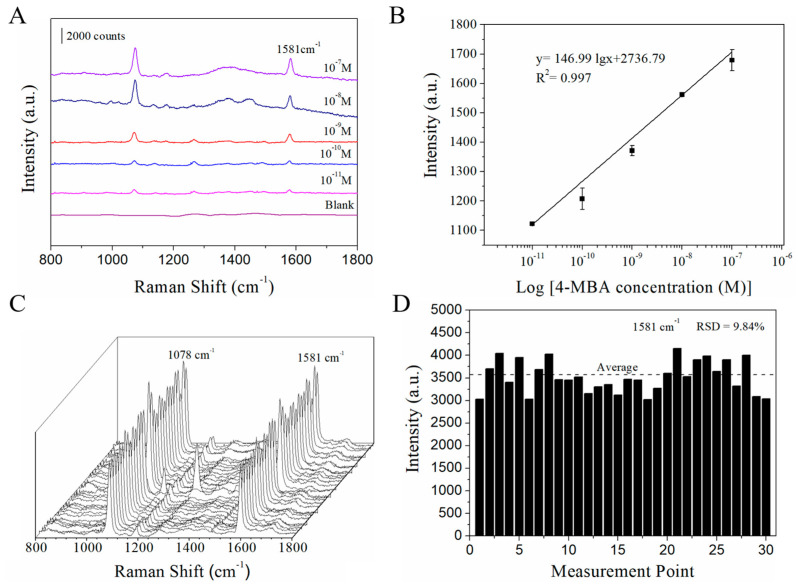
(**A**) SERS spectra detected by Au@Ag NRs substrates for varying concentrations of 4-MBA; (**B**) Linear correlation between SERS intensity at 1581 cm^−1^ and log concentration of 4-MBA; (**C**) SERS spectra of 30 detection points using different Au@Ag NRs substrates; (**D**) SERS intensity distribution of 30 points at 1581 cm^−1^.

Figure 3A showed the SERS spectra using the Au@Ag NRs substrate with different concentrations of 4-MBA. It was observed that the SERS intensity was directly proportional to the logarithm concentration of 4-MBA and the limit of detection (LOD) reached as low as 10^−11^ M. This is significantly lower than the LOD (10^−7^ M) reported by Waiwijit et al. for the detection of 4-MBA on a SERS substrate based on cotton cloth [31]. The results demonstrated the excellent detection sensitivity of the Au@Ag NRs SERS detection platform for 4-MBA. As the strongest SERS intensity was at 1581 cm^−1^, a plot was generated in Figure 3B to depict the relationship between SERS intensity of 4-MBA at 1581 cm^−1^ and the logarithm of its concentration. The fitting regression equation y = 146.99 logx + 2736.79 was obtained with a linearity correlation coefficient (R^2^) of 0.991.

The stability of SERS signals on Au@Ag NRs substrates was assessed using 4−MBA as a probe molecule. Three distinct batches of Au@Ag NRs were selected, and ten points from each batch were randomly tested. As depicted in Figure 3C, two significant Raman peaks were observed at 1078 cm^−1^ and 1581 cm^−1^, respectively. These peaks showed little change in SERS intensity. Furthermore, the SERS intensities at 1581 cm^−1^ are also shown in Figure 3D. The relative standard deviation (RSD) of the 30 SERS intensities at 1581 cm^−1^ was determined as 9.84%. It is generally accepted that the relative standard deviation of SERS substrates between batches or between different test points in the same batch should not exceed 20% [32]. Therefore, the Au@Ag NRs substrate developed in this study demonstrated excellent stability of SERS signals.

### 3.4. Detection Performance Evaluation of Real Samples Based on Au@Ag NRs Substrate

#### 3.4.1. Optimization of SERS Detection for Malachite Green

**Figure 4:**
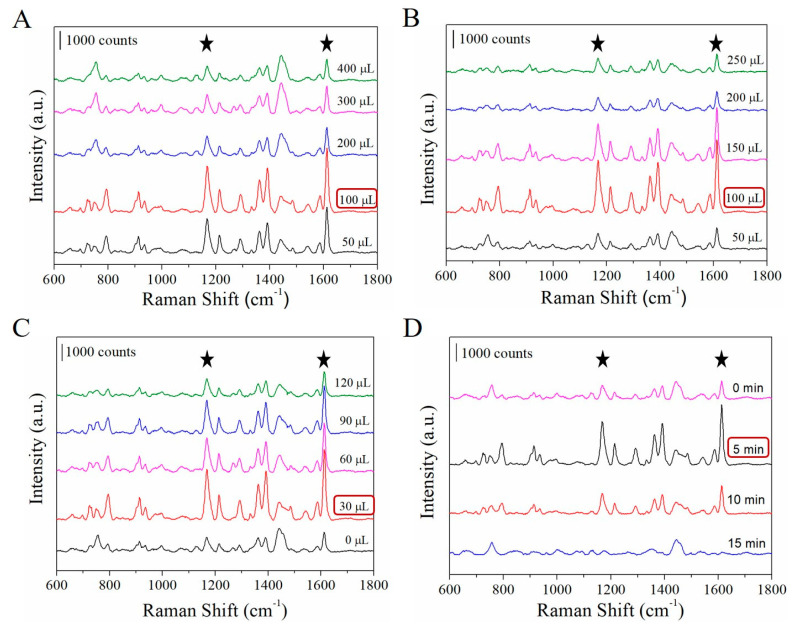
SERS spectra for detection of malachite green using different addition amounts of (**A**) Au@Ag NRs, (**B**) MG and (**C**) 0.1 M NaCl, and (**D**) different incubating time. The black stars denote the SERS intensity of two selected peaks during the optimization process, and the red boxes denote the optimal conditions in detection.

The optimal enhancement of SERS detection is achieved when the analyte’s vibration mode is perpendicular to the substrate, which relies on both the quantity of substrate and analyte added. Therefore, the addition amount of Au@Ag NRs and malachite green (MG) was crucial in SERS detection [33]. In addition, it was found that the aggregation or assembly of Au@Ag NRs would be initiated by the addition of salt, due to the electrostatic neutralization [34]. Therefore, in order to detect the MG in foods based on Au@Ag NRs substrate, for the addition amount of 10^−6^ M MG, Au@Ag NRs substrate and 0.1 M NaCl, the reaction time of the mixture of the above substance were optimized. The highest SERS intensity at 1167 cm^−1^ and 1617 cm^−1^ was found in the 100 μL of Au@Ag NRs substrate (Figure 4A), 100 μL of MG (Figure 4B) and 30 μL of NaCl (Figure 4C), respectively. The optimal reaction time was observed at 5 min (Figure 4D). Therefore, the optimal condition for SERS based on Au@Ag NRs to detect MG was to use 100 μL Au@Ag NRs substrate, 100 μL 10^−6^ M MG and 30 μL 0.1 M NaCl, and the signal was collected after incubation of the mixture for 5 min.

#### 3.4.2. Detection Sensitivity and Accuracy

Under the optimal detection conditions, we investigated the SERS detection sensitivity and accuracy of MG using the developed Au@Ag NRs substrate. In surface-enhanced Raman spectroscopy, the intensity of the characteristic peak corresponding to the analyte in SERS is directly proportional to its concentration. The peak at 1167 cm^−1^ corresponds to the bending vibration of the C-H bond in the benzene ring and the peak at 1617 cm^−1^ corresponds to the stretching vibration of the C-C bond within the benzene ring [35]. During MG determination, we selected the linear relationship between the Raman intensity at 1617 cm^−1^ and the paired value of the MG concentration.

**Figure 5:**
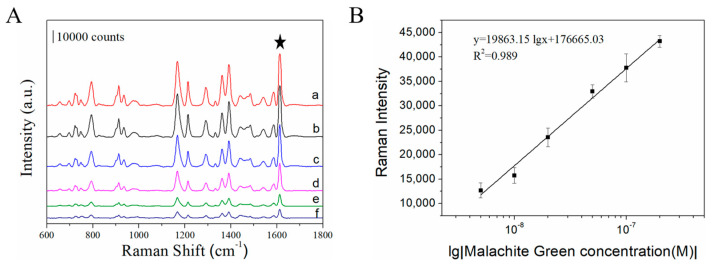
SERS spectra of malachite green at different concentrations using Au@Ag NRs substrate (**A**), and quantitative standard curve of MG based on the SERS intensity at 1617 cm^−1^ (**B**). a: 2 × 10^−7^ M; b: 10^−8^ M; c: 5 × 10^−8^ M; d: 2 × 10^−8^ M; e: 10^−8^ M; f: 5 × 10^−9^ M. The black star refers to utilize the SERS intensity at 1617 cm^−1^ to assess the concentration of MG.

A gradual decline in SERS intensity was observed with decreasing MG concentration in Figure 5A. The fitting regression equation was y = 19,863.15 logx + 176,665.03, the linearity correlation of R^2^ was 0.989 and the limit of detection (LOD) for MG was determined to be 1.58 × 10^−9^ M (Figure 5B). Liu et al. [36] prepared Au@SiO_2_ nanoparticles to detect MG in fish, realizing an LOD of 1.5 × 10^−9^ M. Zhang et al. [33] constructed a nanosphere SERS sensor for the detection of MG in fish with an LOD of 1.37 × 10^−9^ M. Yue X et al. [37] developed a ratio-metric fluorescent sensor combined with a smart phone to achieve MG detection of fish with an LOD of 4.35 × 10^−6^ M. Therefore, the SERS detection sensitivity of MG, based on the developed Au@Ag NRs substrate, was at the same level as the reported methods. However, the developed Au@Ag NRs substrate had the advantages of stable SERS signal, uniform size and low cost, which can be used for the detection of MG in aquatic products.

**Figure 6:**
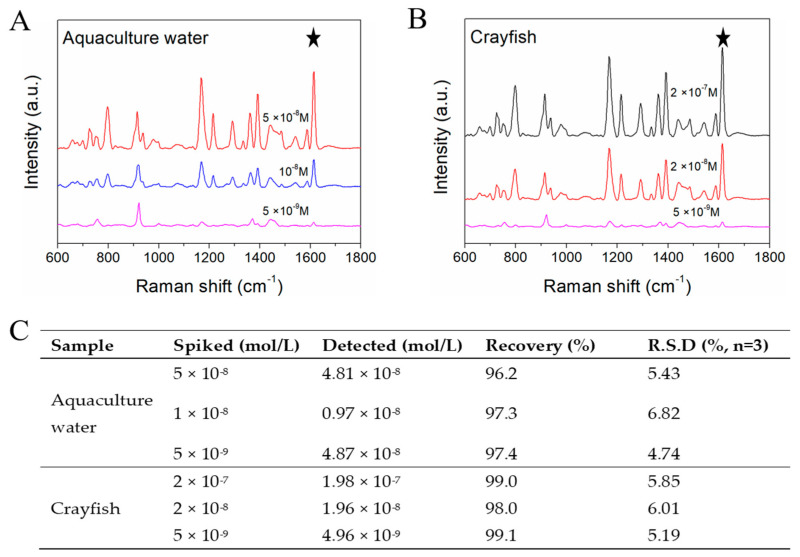
SERS spectra of malachite green in aquaculture water (**A**) and in crayfish (**B**) at different concentrations based on Au@Ag NRs substrate. (**C**) Recovery results of SERS detection for MG in aquaculture water and crayfish. The black star refers to utilize the SERS intensity at 1617 cm^−1^ to assess the concentration of MG.

To evaluate the accuracy of SERS detection of MG by Au@Ag NRs substrate, the spiked MG samples in aquaculture water and crayfish were detected, respectively. The SERS spectra are shown in Figure 6A (aquaculture water) and Figure 6B (crayfish). As shown in Figure 6C, the recovery rate of MG in aquatic water was 89.6~106.0%, and its RSD was 4.74~6.82%. While the recovery rate of MG in crayfish muscle was 93.5~107.0%, with the RSD ranging from 5.19~6.01%. These results indicated the developed Au@Ag NRs sensor had good accuracy for MG detection.

## 4. Conclusions

In this study, Au@Ag NRs substrate was developed with simple preparation and a good SERS enhancement effect. Its SERS signal was 8.7 times higher than that of the AuNRs substrate. The sensitivity for 4-MBA of SERS detection based on Au@Ag NRs substrate was low to 10^−11^ M. In addition, the fabricated Au@Ag NRs substrate had the advantages of stable SERS signal, uniform size and low cost, and could be used for the detection of MG in aquaculture water and crayfish; the limit of detection of MG was calculated as 1.58 × 10^−9^ M. This study provided a new tool for SERS signal enhancement and a highly sensitive SERS detection method development.

## Data Availability

Not applicable.

## References

[1] Zhang W., Ma J., Sun D.-W. (2021). Raman spectroscopic techniques for detecting structure and quality of frozen foods: Principles and applications. Crit. Rev. Food Sci. Nutr..

[2] Zhu J., Jiang X., Rong Y., Wei W., Wu S., Jiao T., Chen Q. (2023). Label-free detection of trace level zearalenone in corn oil by surface-enhanced Raman spectroscopy (SERS) coupled with deep learning models. Food Chem..

[3] Wang Y., Wang M., Shen L., Sun X., Shi G., Ma W., Yan X. (2018). High-performance flexible surface-enhanced Raman scattering substrates fabricated by depositing Ag nanoislands on the dragonfly wing. Appl. Surf. Sci..

[4] Willets K.A., Van Duyne R.P. (2007). Localized surface plasmon resonance spectroscopy and sensing. Annu. Rev. Phys. Chem..

[5] Zheng J., He L. (2014). Surface-enhanced Raman spectroscopy for the chemical analysis of food. Compr. Rev. Food Sci. Food Saf..

[6] Creedon N.C., Lovera P., Furey A., O’Riordan A. (2018). Transparent polymer-based SERS substrates templated by a soda can. Sens. Actuators B Chem..

[7] Yang L., Zhen S.J., Li Y.F., Huang C.Z. (2018). Silver nanoparticles deposited on graphene oxide for ultrasensitive surface-enhanced Raman scattering immunoassay of cancer biomarker. Nanoscale.

[8] Li H., Geng W., Zheng Z., Haruna S.A., Chen Q. (2023). Flexible SERS sensor using AuNTs-assembled PDMS film coupled chemometric algorithms for rapid detection of chloramphenicol in food. Food Chem..

[9] Bai S., Du Y., Wang C., Wu J., Sugioka K. (2019). Reusable surface-enhanced Raman spectroscopy substrates made of silicon nanowire array coated with silver nanoparticles fabricated by metal-assisted chemical etching and photonic reduction. Nanomaterials.

[10] Gill H.S., Thota S., Li L., Ren H., Mosurkal R., Kumar J. (2015). Reusable SERS active substrates for ultrasensitive molecular detection. Sens. Actuators B Chem..

[11] Chien Y.-S., Chang C.-W., Huang C.-C. (2022). Differential surface partitioning for an ultrasensitive solid-state SERS sensor and its application to food colorant analysis. Food Chem..

[12] Nie S., Emory S.R. (1997). Probing single molecules and single nanoparticles by surface-enhanced Raman scattering. Science.

[13] Wu L., Pu H., Huang L., Sun D.-W. (2020). Plasmonic nanoparticles on metal-organic framework: A versatile SERS platform for adsorptive detection of new coccine and orange II dyes in food. Food Chem..

[14] Muhammad M., Yao G., Zhong J., Chao K., Aziz M.H., Huang Q. (2020). A facile and label-free SERS approach for inspection of fipronil in chicken eggs using SiO2@ Au core/shell nanoparticles. Talanta.

[15] Chen C., Wang X., Waterhouse G.I., Qiao X., Xu Z. (2022). A surface-imprinted surface-enhanced Raman scattering sensor for histamine detection based on dual semiconductors and Ag nanoparticles. Food Chem..

[16] Qi X., Wang X., Dong Y., Xie J., Gui X., Bai J., Duan J., Liu J., Yao H. (2022). Fast synthesis of gold nanostar SERS substrates based on ion-track etched membrane by one-step redox reaction. Spectrochim. Acta Part A Mol. Biomol. Spectrosc..

[17] Parambath J.B., Kim G., Han C., Mohamed A.A. (2022). SERS performance of cubic-shaped gold nanoparticles for environmental monitoring. Res. Chem. Intermed..

[18] Columbus S., Hamdi A., Ramachandran K., Daoudi K., Dogheche E.H., Kaidi M. (2022). Rapid and ultralow level SERS detection of ethylparaben using silver nanoprisms functionalized sea urchin-like Zinc oxide nanorod arrays for food safety analysis. Sens. Actuators A Phys..

[19] Liu B., Han G., Zhang Z., Liu R., Jiang C., Wang S., Han M.-Y. (2012). Shell thickness-dependent Raman enhancement for rapid identification and detection of pesticide residues at fruit peels. Anal. Chem..

[20] Chen Z., Sun Y., Shi J., Zhang W., Zhang X., Huang X., Zou X., Li Z., Wei R. (2022). Facile synthesis of Au@ Ag core–shell nanorod with bimetallic synergistic effect for SERS detection of thiabendazole in fruit juice. Food Chem..

[21] Pu H., Huang Z., Xu F., Sun D.-W. (2021). Two-dimensional self-assembled Au-Ag core-shell nanorods nanoarray for sensitive detection of thiram in apple using surface-enhanced Raman spectroscopy. Food Chem..

[22] Xu T., Wang X., Huang Y., Lai K., Fan Y. (2019). Rapid detection of trace methylene blue and malachite green in four fish tissues by ultra-sensitive surface-enhanced Raman spectroscopy coated with gold nanorods. Food Control.

[23] Zhao Q., Lu D., Zhang G., Zhang D., Shi X. (2021). Recent improvements in enzyme-linked immunosorbent assays based on nanomaterials. Talanta.

[24] Pinzaru S.C., Magdas D.A. (2018). Ag nanoparticles meet wines: SERS for wine analysis. Food Anal. Methods.

[25] Ouyang L., Yao L., Zhou T., Zhu L. (2018). Accurate SERS detection of malachite green in aquatic products on basis of graphene wrapped flexible sensor. Anal. Chim. Acta.

[26] ItoItoh T., Uchida T., Izu N., Matsubara I., Shin W. (2017). Effect of Core-shell Ceria/Poly(Vinylpyrrolidone) (PVP) Nanoparticles Incorporated in Polymer Films and Their Optical Properties (2): Increasing the Refractive Index. Materials.

[27] Hang Y., Boryczka J., Wu N. (2022). Visible-light and near-infrared fluorescence and surface-enhanced Raman scattering point-of-care sensing and bio-imaging: A review. Chem. Soc. Rev..

[28] Oliveira M.J., Rubira R.J., Furini L.N., Batagin-Neto A., Constantino C.J. (2020). Detection of thiabendazole fungicide/parasiticide by SERS: Quantitative analysis and adsorption mechanism. Appl. Surf. Sci..

[29] Zhu J., Zhang F., Li J.-J., Zhao J.-W. (2014). The effect of nonhomogeneous silver coating on the plasmonic absorption of Au–Ag core–shell nanorod. Gold Bull..

[30] Chen J., Wei Z., Cao X.-y. (2019). QuEChERS pretreatment combined with ultra-performance liquid chromatography–tandem mass spectrometry for the determination of four veterinary drug residues in marine products. Food Anal. Methods.

[31] Ge F., Ga O.L., Peng X., Li Q., Wang Z. (2020). Atmospheric pressure glow discharge optical emission spectrometry coupled with laser ablation for direct solid quantitative determination of Zn, Pb, and Cd in soils. Talanta.

[32] Sun H., Liu H., Wu Y. (2017). A green, reusable SERS film with high sensitivity for in-situ detection of thiram in apple juice. Appl. Surf. Sci..

[33] Birke R.L., Znamenskiy V., Lombardi J.R. (2010). A charge-transfer surface enhanced Raman scattering model from time-dependent density functional theory calculations on a Ag 10-pyridine complex. J. Chem. Phys..

[34] Awada C., Dab C., Grimaldi M., Alshoaibi A., Ruffino F. (2021). High optical enhancement in Au/Ag alloys and porous Au using Surface-Enhanced Raman spectroscopy technique. Sci. Rep..

[35] Xu N.N., Zhang Q., Guo W., Li Q.T., Xu J. (2016). Au@PVP Core-Shell Nanoparticles Used as Surface-Enhanced Raman Spectroscopic Substrate to Detect Malachite Green. Chin. J. Anal. Chem..

[36] Liu Y., Lei L., Wu Y., Chen Y., Yan J., Zhu W., Tan X., Wang Q. (2022). Fabrication of sea urchin-like Au@ SiO2 nanoparticles SERS substrate for the determination of malachite green in tilapia. Vib. Spectrosc..

[37] Yue X., Li Y., Xu S., Li J., Li M., Jiang L., Jie M., Bai Y. (2022). A portable smartphone-assisted ratiometric fluorescence sensor for intelligent and visual detection of malachite green. Food Chem..

